# IMPROVING HEALTH EQUITY BY ENHANCING ACCESS TO TECHNOLOGY FOR OLDER ADULTS IN RURAL AREAS

**DOI:** 10.1093/geroni/igae098.0916

**Published:** 2024-12-31

**Authors:** Shannon Freeman, Rashelle Hoffman, Rashelle Hoffman

**Affiliations:** Chair; Co-Chair; Discussant

## Abstract

The COVID-19 pandemic generated unprecedented acceleration in the adoption of technology (from digital apps and wearable sensors to robotics and artificial intelligence) in rural areas. However, this rapid digitalization across the care continuum was characterized by inequitable distribution, implementation, and unsustainability in rural healthcare settings. Although health is a fundamental human right, there are many dimensions of inequality, including sex, gender, ethnicity, and disability, that are amplified in rural healthcare settings. This symposium will showcase how research has supported the identification, implementation, and sustainment of innovative technology solutions to enhance health equity in rural settings (Freeman). With the cumulative shift to virtual delivery of health care services, there is need to ensure equitable access to infrastructure and resources to address existing technology gaps which disproportionally affect older adults living in rural communities (Clayton-Jones). Additionally, with the expanding breadth of technology applications being used by older adults, this symposium will showcase the positive technological impacts such as autonomous vehicles (Yoon), smartwatches (Wiese), and eReaders (Agboji) to enhance health equity for older adults. Rural Aging Interest Group Sponsored Symposium

